# An autoimmune disease risk SNP, rs2281808, in *SIRPG* is associated with reduced expression of SIRPγ and heightened effector state in human CD8 T-cells

**DOI:** 10.1038/s41598-018-33901-1

**Published:** 2018-10-18

**Authors:** Sushmita Sinha, Nicholas Borcherding, Pranav S. Renavikar, Michael P. Crawford, Eva Tsalikian, Michael Tansey, Ezzatollah T. Shivapour, Frank Bittner, John Kamholz, Heena Olalde, Emilee Gibson, Nitin J. Karandikar

**Affiliations:** 1 Department of Pathology, University of Iowa, 200 Hawkins Dr., Iowa City, IA 52242 USA; 2 Department of Pediatrics, University of Iowa, 200 Hawkins Dr., Iowa City, IA 52242 USA; 3 Department of Neurology, University of Iowa, 200 Hawkins Dr., Iowa City, IA 52242 USA

## Abstract

Multiple GWAS studies have shown that the SNP rs2281808 TT variant, present within the *SIRPG* gene, is associated with autoimmune diseases, such as type 1 diabetes. However, the role of SIRPγ in human T-cells is not known, neither is the functional significance of TT variant. Here we investigated *SIRPG* genotypes and their effects on the fate and function of human T-cells. We found that the presence of T variant resulted in reduction of SIRPγ expression on T-cells. Functionally, SIRPγ^low^ CD8 T-cells in CT and TT individuals existed in a heightened effector state with lower activation threshold and had greater expression of genes and molecules associated with migratory and cytotoxic potential. Further, SIRPγ^low^ CD8 T-cells were deficient in transcription factors associated with long-term functional memory formation. Our study reveals biological consequences of the SNP rs2281808 and provides novel insights into the potential mechanisms by which SIRPγ might regulate human immune responses.

## Introduction

Genome-wide association studies have been instrumental in identifying genetic risk variants in autoimmune diseases. However, in most cases, the biological interpretation of how the reported risk variants potentiate autoimmunity remains unknown. Multiple GWAS studies have shown that the SNP rs2281808 TT variant is associated with type 1 diabetes (T1D)^1–3^. Rs2281808 TT is an intronic SNP present between exons 5 and 6 of the Signal Regulatory Protein (*SIRPG*) gene and causes a C/T variant. The rs2281808 T risk allele confers a 1.1 (1.01–1.19) increased risk of developing T1D (per dbSNP; https://www.ncbi.nlm.nih.gov/projects/SNP/) and might be associated with early onset diabetes patients^3^. The biological consequence of rs2281808 T variant and the mechanisms by which it potentiates autoimmunity remains unknown.

SIRPγ is absent in the rodents. In humans, SIRPγ is the only SIRP expressed by T-cells^4^ and its function is poorly understood. Engagement of SIRPγ on T cells by CD47 on APCs has been shown to enhance antigen-specific T-cell proliferation^5^. SIRPγ may also be required for trans-endothelial migration of human T-cells *in vitro*^6^. The most substantial hint for the mechanistic role of SIRPγ in autoimmunity comes from a recent study predicting that polymorphisms in *SIRPG* gene can interfere with transcription factors important in T-cell development^7^. Further, Differential expression of *SIRPG* has also been reported in Systemic Lupus Erythematosus (SLE) patients, suggesting that SIRPγ might be pathologically relevant in multiple autoimmune diseases. Since polymorphism in *SIRPG* gene is associated with the development of T1D, we hypothesized that the rs2281808 genotype might modulate SIRPγ-mediated regulation of T-cell effector responses.

We provide the first evidence that rs2281808 T variant is associated with a reduction in SIRPγ expression on human T-cells and that this can have potentially pathogenic consequences since SIRPγ^low^ CD8 T-cells were characterized by exaggerated effector responses.

## Results

### SNP rs2281808 TT is associated with the reduction of SIRPγ expression on T cells

**Figure 1:**
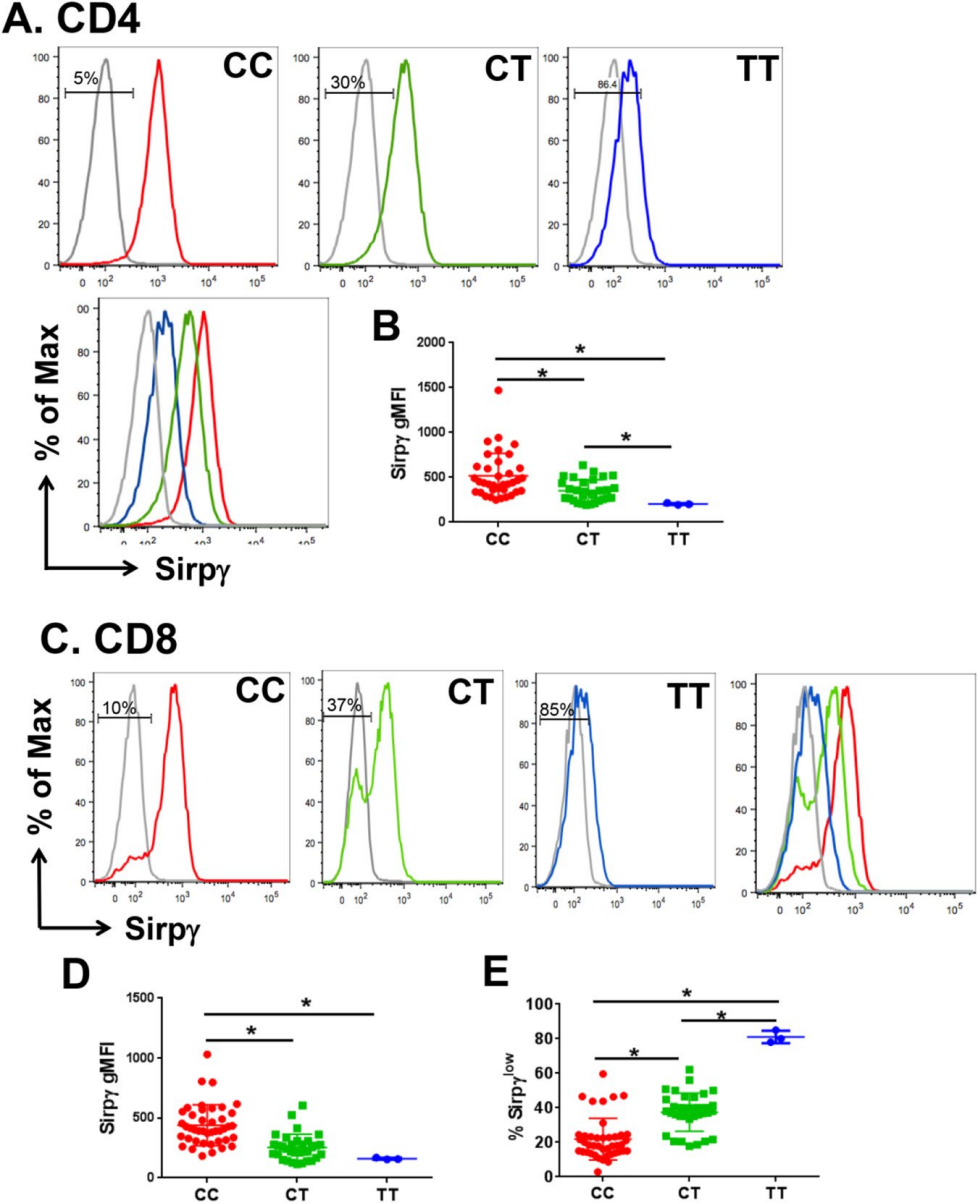
Autoimmune disease risk SNP rs2281808 causes low of SIRPγ expression on human T-cells. All the 79 PBMC samples from HD were subjected to flow cytometry staining and genotyping for rs2281808 using TaqMan chemistry. SIRPγ expression relative to rs2281808 genotyping status was analyzed on gated CD3 CD4 and CD3 CD8 T-cells (**A**–**E**). Representative histograms (**A**,**C**) and cumulative MFI data (**B**,**D**) are shown. CD8 T-cells showed a bimodal expression of SIRPγ, which was used to determine the frequency of SIRPγ^high^ and SIRPγ^low^ cells. The frequency of SIRPγ^low^ CD8 T-cells is shown in (**E**). Isotype staining is shown in grey. Gates are shown for SIRPγ^low^ cells. One-way ANOVA with Tukey’s posthoc test was performed and p < 0.05 was considered significant.

To determine whether the rs2281808 TT variant regulates SIRPγ expression on T-cells, 79 healthy donors (HD) were genotyped for SNP rs2281808 and assessed for SIRPγ expression. We found that 45 and 31 HD showed the CC and CT genotypes, respectively, whereas the TT variant was present in 3 HD. Flow cytometry revealed that the CC genotype was associated with robust SIRPγ expression on the majority of CD4 and CD8 T-cells. In contrast, CD4 (Fig. 1A,B) and CD8 (Fig. 1C,D) T-cells from rs2281808 TT carriers had significantly reduced surface expression of SIRPγ, whereas the CT genotype was associated with an intermediate SIRPγ expression that was significantly lower than CC cells (SIRPγ-MFI on CD4 T-cells in TT vs. CT vs CC: 203 ± 10.8 vs. 350 ± 123 vs 526 ± 244, CC vs. CT & CT vs. TT, p < 0.05; CC vs. TT, p < 0.01, p < 0.05 and SIRPγ-MFI on CD8 T-cells in TT vs. CT vs. CC: 160 ± 7.9 vs. 275 ± 93 vs. 439 ± 170; CC vs. CT & CT vs. TT, p < 0.05; CC vs. TT, p < 0.01).

We also noted that, in contrast to the unimodal distribution of SIRPγ on CD4 T-cells, it showed a bimodal distribution on CD8 T-cells, which was particularly pronounced in CT carriers (Fig. 1C), who showed significantly greater frequencies of SIRPγ^low^ CD8 T-cells as compared to CC carriers (21.8% ± 12 vs. 37.4% ± 11, p < 0.05; Fig. 1E). In keeping with the MFI, the majority of CD8 T-cells (≥80%) in TT carriers were SIRPγ^low^ (Fig. 1C,E).

### Unlike CT/TT carriers, SIRPγ^low^ CD8 T-cells in CC carriers are absent from the naïve pool

We also noted that 6/42 (14%) of CC individuals in HD showed relatively higher frequencies of SIRPγ^low^ CD8 T-cells compared to the rest of the CC donors. Similarly, there were 7/32 (22%) of CT individuals who showed a relatively low fraction of SIRPγ^low^ CD8 T-cells (Fig. 1E, outliers). In this regard, the CC individuals exhibited a CT pattern of staining and vice versa. We hypothesized that the SIRPγ^low^ cells from CC individuals may represent downregulation of SIRPγ during effector/memory differentiation (as opposed to having a SIRPγ^low^ fraction in naïve CD8 T-cells). To test this, we evaluated the distribution of SIRPγ-high and low cells within the naïve vs. effector/memory fractions. The gating strategy is shown in Supplementary Fig. 1. The overall distribution of CD8 T cell subsets based on their rs2281808 genotyping status is shown in Supplementary Fig. 2.

**Figure 2:**
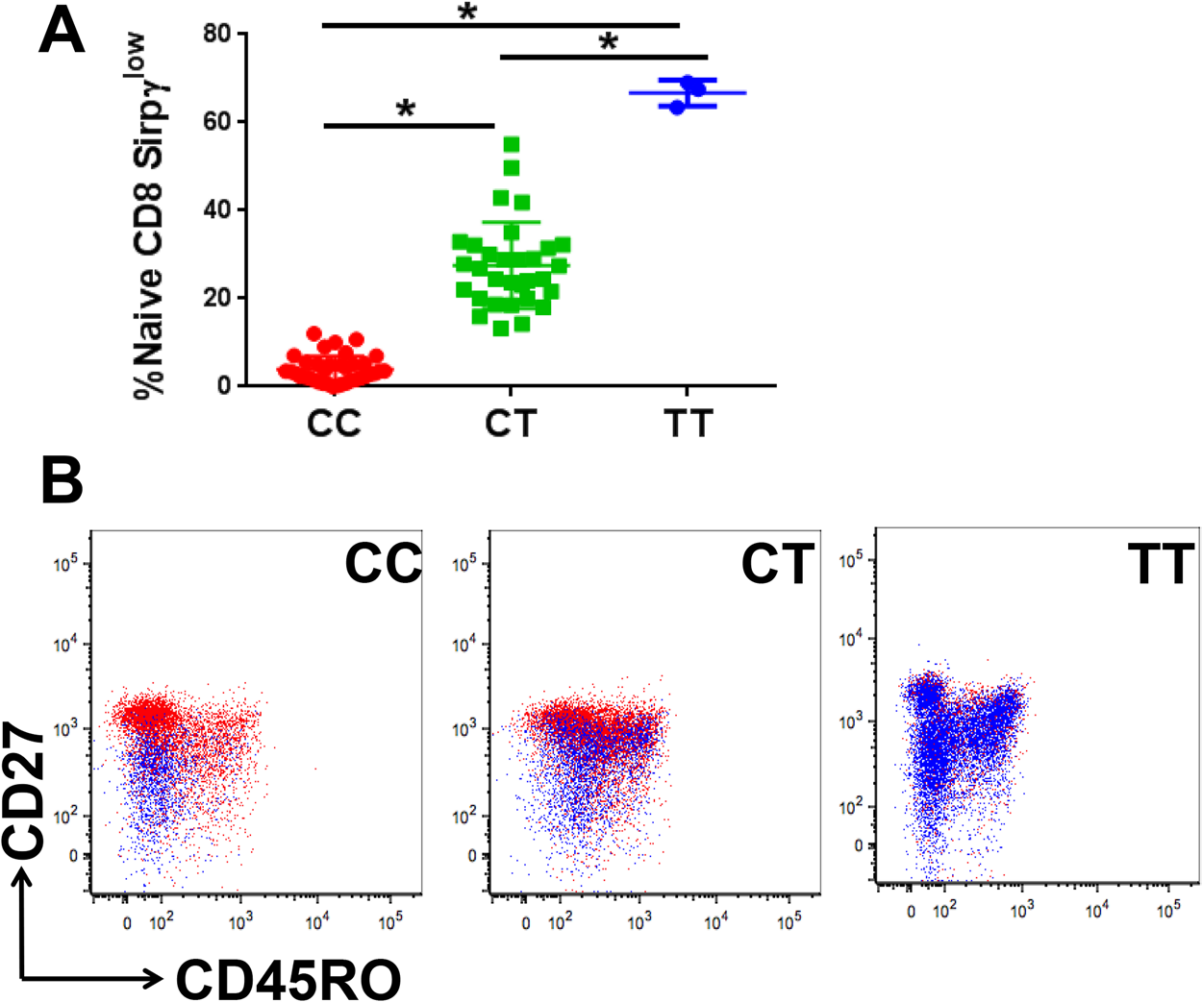
CT and TT carriers, but not CC individuals, have a significantly greater proportion of naïve CD8 T-cells with a SIRPγ^low^ phenotype. Flow cytometry staining for CD27 and CD45RO was used to analyze SIRPγ^low^ CD8 T-cells in the naïve (CD27+ CD45RO−), central memory (CD27+ CD45RO+), effector memory (CD27−CD45RO+) and terminally differentiated CD27−CD45RO−) fractions. Detailed gating strategy is shown in Supplementary Fig. 1. In CC individuals, SIRPγ^low^ CD8 T-cells were essentially absent from the naïve pool, whereas in CT and TT carriers, SIRPγ^low^ CD8 T-cells were present in the naïve pool at increasing frequencies (**A**). Representative dot plots are shown in (**B**), where the blue population indicates SIRPγ^low^ CD8 T-cells and the red population corresponds to the SIRPγ^high^ CD8 T-cells. One-way ANOVA with Tukey’s posthoc test was performed and p < 0.05 was considered significant.

Interestingly, in all CC carriers, the vast majority (94.5% ± 3.4) of SIRPγ^low^ CD8 T-cells were present in memory/terminally-differentiated fraction (Fig. 2A,B). In contrast, SIRPγ^low^ CD8 T-cells from CT carriers were also present at greater proportions in the naïve T-cell fraction (27.5% ± 9.7; Fig. 2A,C). The absence of SIRPγ^low^ CD8 T-cells from the naïve pool of CC carriers suggests that thymically-derived CD8 T-cells of CC individuals express high levels of SIRPγ, which can then downregulate its expression during differentiation. Importantly, C/T replacement even in one locus appears to alter this pattern in CT carriers, where higher frequencies of naïve cells are SIRPγ^low^, significantly more than CC subjects (Fig. 2A). As expected from the overall staining pattern, TT individuals carried SIRPγ^low^ CD8 T-cells in all fractions.

### SIRPγ^low^ CD8 T-cells from CT carriers display an effector cell profile with enhanced cytotoxic potential

**Figure 3:**
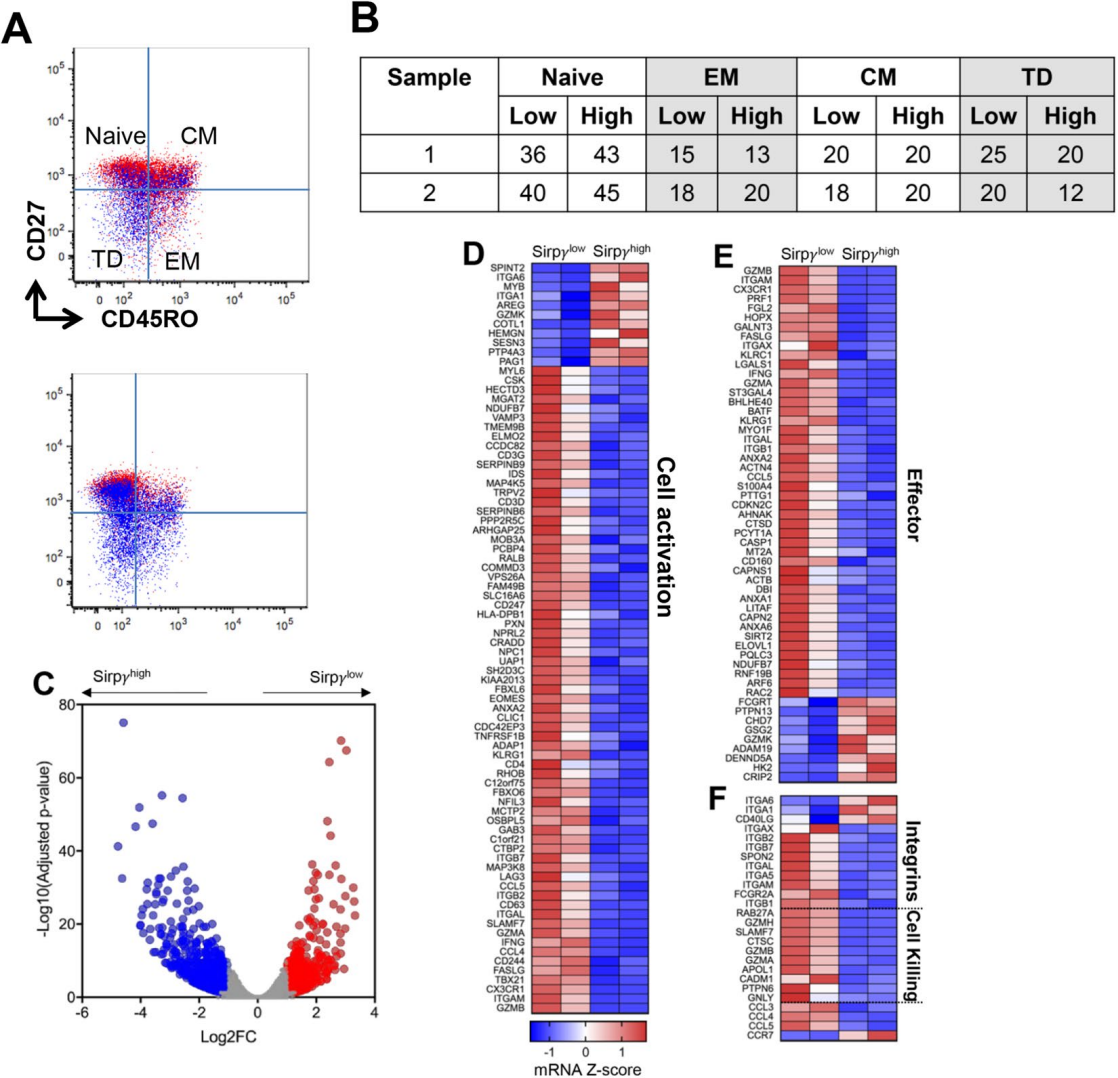
SIRPγ^low^ CD8 T-cells from CT carriers display an effector cell profile with enhanced cytotoxic potential. SIRPγ^low^ and high CD8 T-cells were sorted from two CT carriers and subjected to RNA sequencing (A). SIRPγ^low^ cells are shown in blue (**A**). The distribution of CD8 T-cells between naïve, central memory (CM), effector memory (EM) and terminally differentiated (TD) fraction in the two sorted samples is shown in table (**B**). Volcano plot is comparing SIRPγ^low^ versus SIRPγ^high^ cells with upregulated genes in red and downregulated genes in blue (**C**). Heat maps of SIRPγ^low^ versus SIRPγ^high^ CD8 T-cells are shown. SIRPγ^low^ CD8 T-cells have significant enrichment of genes associated with effector functions, cell activation, cell-cell interaction, and cytotoxicity (**D**–**F**).

**Figure 4:**
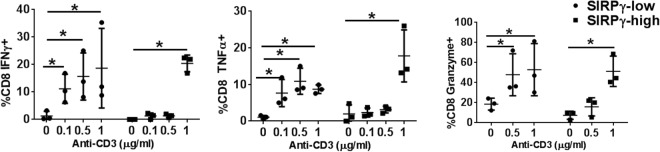
SIRPγ^low^ CD8 T-cells have a lower activation threshold. PBMC samples from CT carriers were stimulated with varying concentration of anti-CD3, followed by intra-cellular flow cytometric analysis to detect cytokine production by SIRPγ^low^ vs. SIRPγ^high^ gated subsets. SIRPγ^low^ CD8 T-cells responded to sub-optimal (lower concentrations) anti-CD3 stimulation by producing significantly greater levels of effector cytokines and granzyme (**A**). SIRPγ^high^ CD8 T-cells responded to only optimum (higher concentration) anti-CD3 stimulation (**B**). 2-way ANOVA with Tukey’s posthoc test was performed and p < 0.05 was considered significant.

We focused on studying CD8 T-cells since the bimodal distribution of SIRPγ on CD8 T-cells gave us the opportunity to study SIRPγ^high^ and SIRPγ^low^ cells from the same HD in subsequent studies, in addition to comparing CC vs. TT CD8 T-cells. First, we performed RNA sequencing analysis on flow-sorted SIRPγ-high vs. low CD8 T-cells from the same individuals. The SIRPγ^low^ versus SIRPγ^high^ cells were equally distributed between naïve, central memory (CM), effector memory (EM) and terminally differentiated (TD) fractions in the two individuals as shown in Fig. 3A and quantified in the table (Fig. 3B). Comparing SIRPγ^low^ versus SIRPγ^high^ CD8 T cells, we observed 399 genes significantly upregulated and 593 genes downregulated, as defined as genes with a log2 fold-change ≥1 and an adjusted p-value < 0.05 (Fig. 3C). We observed that SIRPγ expression could compartmentalize CD8 T-cells into strikingly distinct phenotypic and functional populations (Fig. 3D–F). SIRPγ^low^ CD8 T-cells were enriched for genes associated with effector CD8 T-cells, including several integrins, granzymes, and *IFN-γ* (Fig. 3E). Interestingly, MAP3K8, a serine/threonine kinase selectively expressed by effector CTLs in humans^8^, was significantly elevated in SIRPγ^low^ CD8 T-cells. *Ex vivo*-sorted SIRPγ^low^ CD8 T-cells (in the absence of *in vitro* stimulation) displayed an activated CD8 T-cell-signature, expressing several genes that are upregulated following CD3-CD28 stimulation, including *T-bet*, *EOMES*, integrins, granzymes, *CD244*, *CD247*, *SLAMF7*, *CCL5*, *CCL4*, *IFN-γ* (Fig. 4D). Lower expression of *CCR7* and higher expression of integrins and chemokines (*CCL3*, *CCL4*, *CCL5*) in SIRPγ^low^ cells suggested a phenotype that can enter circulation, infiltrate non-lymphoid organs, establish cellular communications and amplify inflammation (Fig. 3F).

### SIRPγ^low^ CD8 T-cells have a lower activation threshold

To further understand the functional relevance of this profile, PBMC samples from HD were stimulated with varying concentrations of anti-CD3 and CD8 T-cell effector responses were assessed. While SIRPγ^high^ CD8 T-cells responded to only optimal anti-CD3 stimulation (1μg/ml), SIRPγ^low^ CD8 T-cells responded even to sub-optimal anti-CD3 stimulation (0.1μg/ml) by producing significantly more IFN-γ, TNF-α, and granzyme (Fig. 4). Thus, SIRPγ^low^ status conferred easier activation potential.

### CD8 T-cells from TT carriers display the same phenotypic and functional profile as SIRPγ^low^ CD8 T-cells from CC and CT carriers

**Figure 5:**
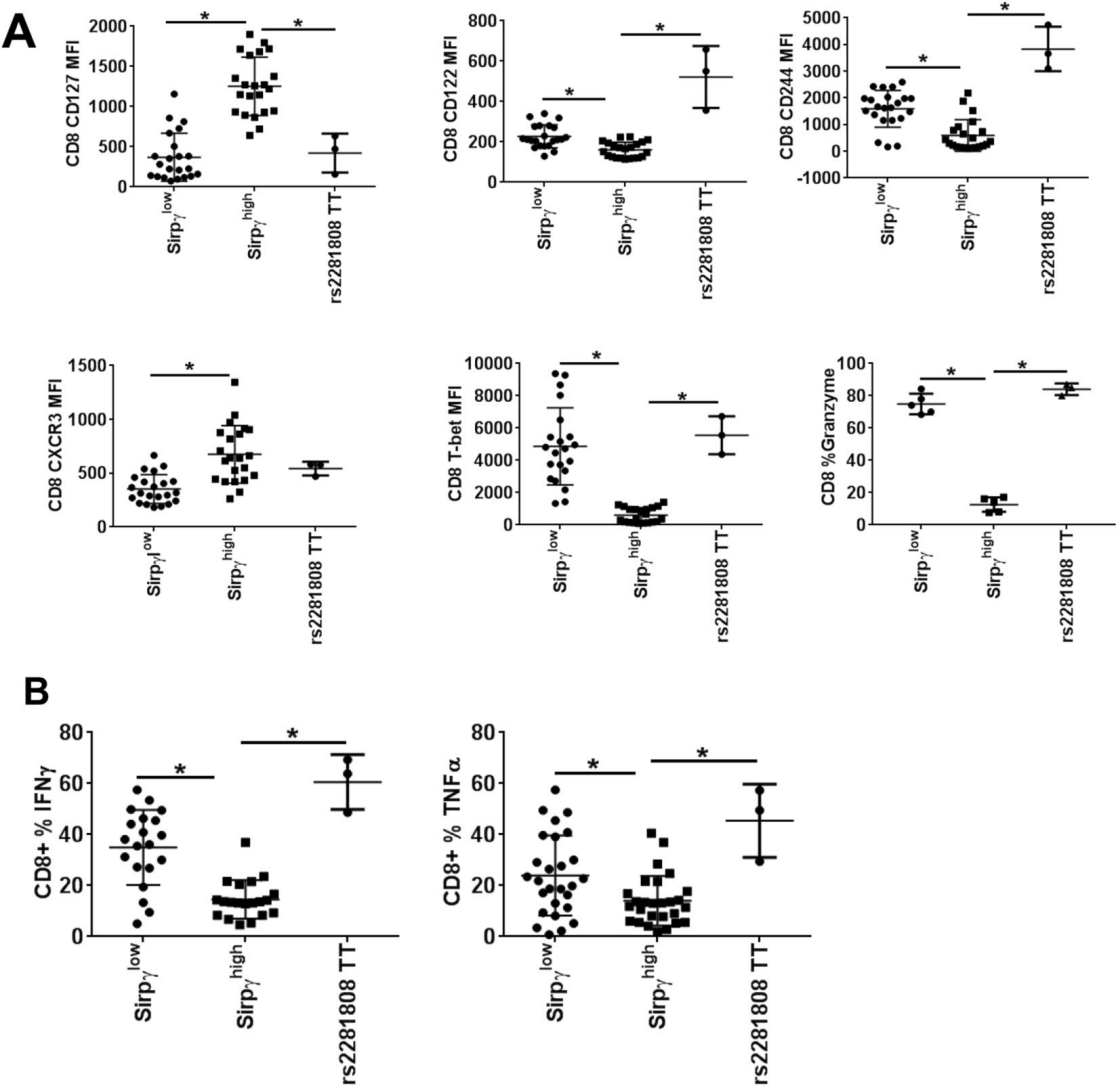
CD8 T-cells from TT carriers display the same phenotypic and functional profile as SIRPγ^low^ CD8 T-cells from CC and CT carriers. (**A**) CD8 T-cells from CC, CT and TT carriers were evaluated for their expression of the indicated molecules by flow cytometry. Intracellular staining was performed for T-bet. In the case of CC and CT, cells were gated for SIRPγ-high vs. low cells. (**B**) PBMC samples from CC, CT, TT carriers were stimulated with PMA/Ionomycin/brefeldin A for five hrs before intracellular staining for IFN-γ and TNF-α. CD8 T-cells from CC and CT carriers were gated on SIRPγ^low^ vs. high cells and evaluated for frequency of cells producing the indicted cytokines. 1-way ANOVA with with Tukey’s posthoc test was performed and p < 0.05 was considered significant.

We next asked whether this RNA signature corresponded to phenotypic and functional consequences, not only in SIRPγ^high^ vs. SIRPγ^low^ CD8 T-cells from the same individual (where the differences may simply reflect different proportions of naïve vs. effector/memory populations) but also in SIRPγ^low^ cells that characterized the TT genotype state in HD. We observed that SIRPγ^low^ CD8 T-cells, whether they were all CD8 T-cells from TT individuals or cells from CT/CC individuals, exhibited features that were similar to each other and, in turn, distinct from SIRPγ^high^ CD8 T-cells. They were characterized by a lower expression of CD127 and higher expression of CD122, CD244, CXCR3, T-bet, and granzyme (Fig. 5A). Functionally, SIRPγ^low^ CD8 T-cells from CC, CT and TT donors produced significantly more IFN-γ and TNF-α on PMA/Ionomycin stimulation as compared to SIRPγ^high^ CD8 T-cells (Fig. 5B). Data from CC and CT carriers separately is shown in Suppl Fig. 3.

### SIRPγ^low^ T cells might be impaired in long term “functional” memory formation

**Figure 6:**
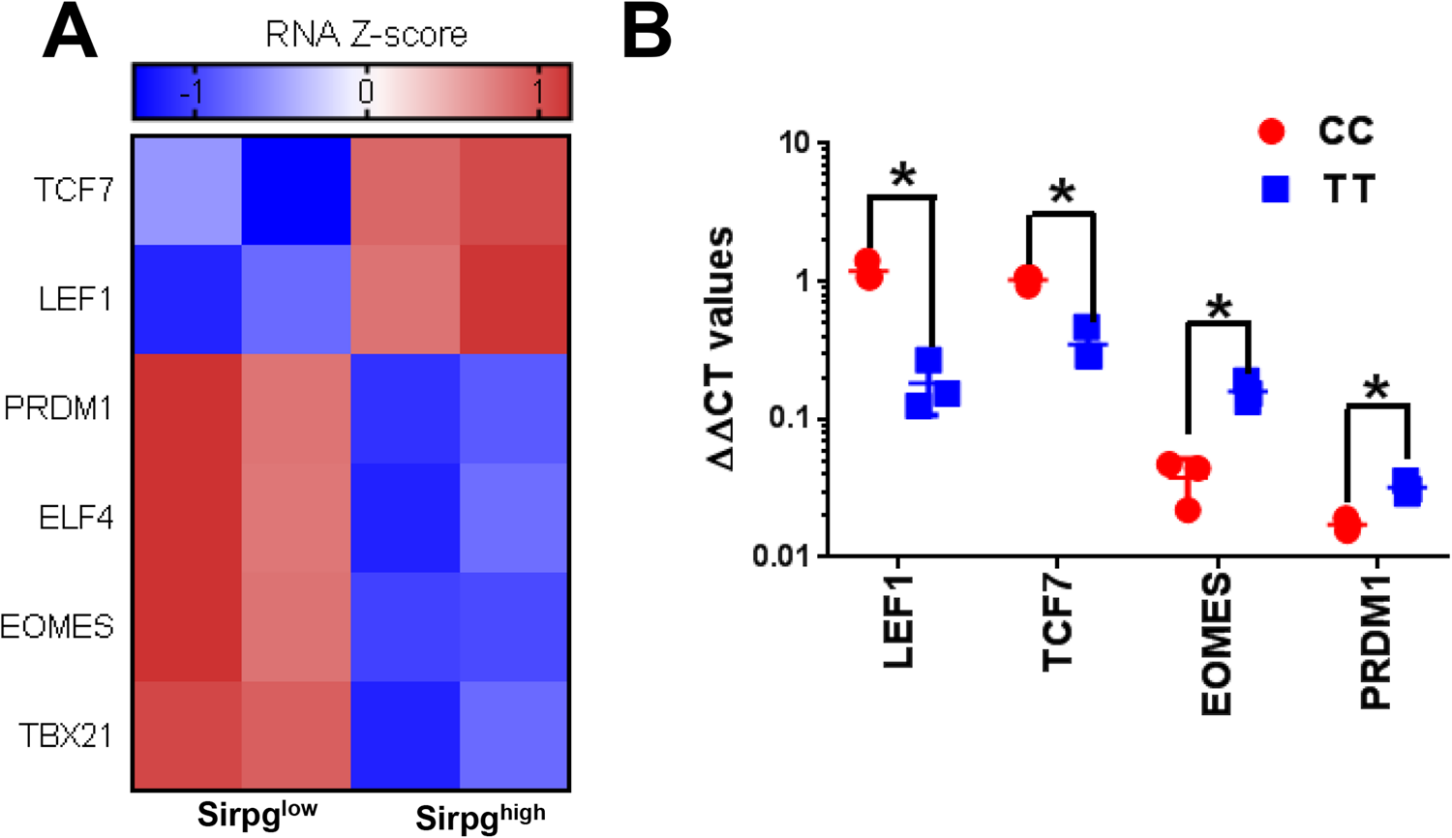
Expression of effector vs. memory-associated transcription factors in SIRPγ^low^ versus SIRPγ^high^ CD8 T-cells. Transcription factor analysis was performed on RNA sequencing data presented in Fig. 3. For RNA sequencing, SIRPγ^low^ and SIRPγ^high^ CD8 T-cells were FACS sorted from two CT carriers and subjected to RNA sequencing. Heat maps of SIRPγ^low^ versus SIRPγ^high^ CD8 T-cells are shown (**A**). SIRPγ^low^ CD8 T-cells have significant downregulation of transcription factors associated with functional memory formation and are enriched in transcription factors upregulated in effector CD8 T-cells (**A**). The transcription factor profile was confirmed in CD8 T-cells from three TT carriers vs. CC carriers by quantitative polymerase chain reaction (qPCR) (**B**). The y-axis shows delta-delta Ct values, after transformation to a logarithmic scale for LEF1, TCF7, EOMES, and PRDM1 in CC vs. TT CD8 T-cells. T-test was performed and p < 0.05 was considered significant.

We then examined the underlying mechanisms that might maintain SIRPγ^low^ CD8 T-cells in effector state. RNA sequencing on sorted SIRPγ^low^ vs. SIRPγ^high^ CD8 T-cells from 2 HDs, as described earlier, suggested that effector T-cell-associated transcription factors, including *T-bet*, *EOMES* and *PRDM1*, were significantly overexpressed in SIRPγ^low^ CD8 T-cells (Fig. 6A). In humans, increased frequency of *EOMES* positive CD8 T-cells is present in the effector populations^9,10^. In CD8 T-cells, PRDM1/BLIMP1 is essential for optimal effector functions including effector cell migration and granzyme production^11–13^. Importantly, expression of two key transcription factors that are required for a long-term functional memory formation, *TCF7* and *LEF1*^14,15^, was significantly downregulated in SIRPγ^low^ CD8 T-cells from CT carriers (Fig. 6A). To study whether this mechanism was also operative in TT carries in HD, gene expression of *TCF7*, *LEF1*, *EOMES*, and *PRDM1* was studied in CD8 T-cells from 3 TT vs. CC carriers by TaqMan real-time PCR. As compared to CC carriers, CD8 T-cells from TT carriers had significantly reduced expression of *LEF1* and *TCF7* expression, respectively (Fig. 6B). Conversely, *EOMES* and *PRDM1* expression were significantly upregulated in CD8 T-cells from TT carriers as compared to CC carriers (Fig. 6B).

## Discussion

Genome-wide association studies have been instrumental in identifying several genetic risk factors associated with autoimmune diseases. However, the biological relevance of many of these mutations/polymorphisms remains understudied and largely unknown. To the best of our knowledge, this study provides the first mechanistic insights into how the single nucleotide polymorphism, rs2281808, might potentiate autoimmunity. Multiple GWAS studies have identified rs2281808 TT variant as a genetic risk factor for type 1 diabetes (T1D), including a study that performed a meta-analysis on combined samples from UK and USA^1^. When associations of the known 21 T1D risk loci with age of diabetes onset were evaluated, the early onset patients provided more association evidence for rs2281808 (p = 0.0015)^3^. We have also found a significantly higher preponderance of TT variant in T1D subjects as compared to healthy donors (HD vs. T1D, 4% vs. 22%; data not shown). Further, our unpublished findings show significantly greater incidence of T allele (CT + TT) in relapsing-remitting multiple sclerosis subjects (data not shown). Collectively, this suggests that SIRPγ might be pathologically relevant in multiple autoimmune diseases. In line with this, the differential expression of *SIRPG* has also been reported in Systemic Lupus Erythematosus (SLE) patients^16^. Despite the accumulating evidence connecting *SIRPG* with autoimmunity, there is a total knowledge gap about the role of SIRPγ in human T-cells and the functional significance of the TT variant. The most substantial hint for the mechanistic role of *SIRPG* in autoimmunity comes from a recent study predicting that polymorphisms in *SIRPG* gene can interfere with transcription factors important in T-cell development^7^.

In this study, we show a pathogenic-effector like phenotype of CD8 T-cells that appears to be genetically defined by the SNP rs2281808 present in the *SIRPG* locus and is independent of their differentiation status. We discovered that autoimmune disease risk SNP rs2281808 is associated with a reduction in SIRPγ expression on T-cells in humans. SNP rs2281808 has also been identified as eQTL in thymus^7^. Our results are in accordance with the GTEx whole blood meta-analysis which shows this SNP as being negatively associated with SIRPγ expression. (https://www.gtexportal.org/home/eqtls/bySnp?snpId=rs2281808&tissueName=All). Mechanistically, this might have a major impact on the effector responses from CD8 T-cells since reduction in SIRPγ expression on CD8 T cells 1) leads to potentiation of effector responses including cytokine secretion, and increased expression of genes and molecules associated with adhesion and cytotoxicity 2) is associated with their lower activation threshold and 3) is associated with imbalance in transcription factors required for functional memory formation vs. factors that stay upregulated in effector CD8 T-cells. Due to the enhanced effector responses displayed by SIRPγ^low^ CD8 T-cells, we propose that their increased frequency, as determined by rs2281808 genotyping status, might lead to exaggerated immune responses and predisposition to autoimmunity.

Our study gives the first insights into the functional consequences in CD8 T-cells associated with their SIRPγ expression. HDs displayed distinct SIRPγ expression pattern on T-cells which was determined by rs2281808 genotyping: homozygous CC carriers have high SIRPγ expression on >80% T cells; heterozygous CT carriers have greater frequency (25–50%) of SIRPγ^low^ T-cells; and finally homozygous TT carriers who have lost SIRPγ expression on >80% T-cells. Additionally, our data also suggest that SIRPγ expression on CD8 T-cells appears to be susceptible to other cell intrinsic and/or extrinsic factors during differentiation. In CC individuals, naïve CD8 T-cells maintain high SIRPγ expression, whereas SIRPγ^low^ CD8 T-cells are confined to memory/terminally differentiated cells. The SNP rs2281808 can cause an imbalance in the ratio of SIRPγ^low^ vs. SIRPγ^high^ CD8 T-cells in CT/TT individuals leading to detrimental functional consequences as explained below.

We find it quite striking that just based on SIRPγ expression alone, human CD8 T-cells can be compartmentalized into distinct phenotypic and functional populations in humans. As compared to their high counterparts, SIRPγ low CD8 T-cells had an effector cell phenotype and responded to suboptimal TCR stimulation. This might be pathologically relevant in autoimmunity since self-reactive T-cells do respond to lower doses of antigens raising an interesting possibility that self-reactive T-cells might predominantly be present in SIRPγ^low^ fraction. In fact, multiple features of SIRPγ^low^ CD8 T-cells, as revealed by our RNA sequencing and functional studies, show that SIRPγ^low^ CD8 T-cells are armed with tissue-damaging effector responses positioning them as “the” pathogenic cells in proinflammatory diseases. SIRPγl^ow^ CD8 T-cells are maintained in heightened effector state with reduced ability to form long term functional memory cells. Based on the expression studies, SIRPγ^low^ CD8 T-cells appear to be better at infiltrating non-lymphoid organs and establishing cellular communications than their high counterparts. Low expression of CCR7 on SIRPγ^low^ CD8 T-cells would enable them to enter circulation and infiltrate into tissues under inflammatory conditions. The infiltration process might be further facilitated by integrins, several of which had a significantly higher expression in SIRPγ^low^ CD8 T-cells. Further, these cells might be more potent in attracting other cells to amplify inflammation as several chemokine ligands, including CCL3, CCL4, CCL5, had significantly increased message in SIRPγ^low^ CD8 T-cells. Finally, significantly higher expression of granzyme B (as shown by FACS) and elevated message for several other granzymes (as shown by sequencing) would suggest that SIRPγ^low^ CD8 T-cells have greater cytotoxic potential as compared to their high counterparts. Of pathogenic relevance, these findings present a unique conundrum for SIRPγ^low^ naïve CD8 T-cells in CT/TT individuals, as they are poised to function as potentiated effector cells irrespective of their naïve status.

Interestingly, our study shows that SIRPγ^low^ CD8 T-cells have a unique transcriptional profile (Tbet high, EOMES high, PRDM1/BLIMP1 high) that enables them to stay in an effector state. In fact, we observed the significantly higher frequency of effector memory CD8 T-cells in TT carriers vs. CC carriers (Suppl Fig. 2). Of note, heightened T-bet expression was a striking feature of SIRPγ^low^ CD8 T-cells (Fig. 5A). High T-bet expression in CD8 T-cells induces short-lived effector cells over memory precursor effector cells^17^. Studies in murine models have shown that certain inflammatory cues regulate T-bet expression in CD8 T-cells^17^. A tight inverse correlation between SIRPγ and T-bet expression in humans raises an interesting possibility whether SIRPγ might be one of the players that, with other inflammatory signals, might regulate T-bet expression in CD8 T-cells and subsequent effector vs. memory fate decision. This dynamic brings into focus the SNP rs2281808 that regulates expression of SIRPγ on T-cells. In fact in rs2281808 TT carriers, >80% CD8 T-cells had low expression of SIRPγ and high expression of T-bet. A recent study predicted that rs2281808 might interfere with the binding of transcription factors implicated in T-cell development^7^. It is tempting to speculate that this maybe the case with T-bet and remains an interesting question to be explored in future studies.

The mechanism by which this intronic SNP rs2281808 regulates SIRPγ expression on T-cells remains unknown. One possibility is that rs2281808 might generate an alternative splice variant of *SIRPG*, leading to apparent loss of protein. Alternatively, spliced transcript variants encoding different isoforms of *SIRPG* have been described. It is possible that there might be another isoform of SIRPγ that is not recognized by this antibody.

Functional effects that we see on CD8 T-cells could be mediated indirectly through other molecules. *SIRPG* has also been reported to regulate the expression of *SIRPD* and *NSFL1C* in trans-eQTL manner^18^. SIRPD is not expressed by human T-cells. NSFL1C is an intracellular ATPase involved in transport vesicle/target membrane fusion and fusions between membrane compartments.

Overall, our study provides novel mechanistic insights into how SNP rs2281808 TT variant might predispose individuals to immune-mediated diseases like T1D. Our study suggests that the frequency of SIRPγ^low^ CD8 T-cells can determine the magnitude and quality of immune response. Increased frequencies of SIRPγ^low^ CD8 T-cells, as determined by the rs2281808 T allele, would result in exaggerated effector responses from CTLs which could then contribute to tissue pathology. Interestingly, TT or CT status can negatively impact functional memory formation in CD8 T-cells. This can potentially have a significant impact on the overall fate of the immune response, including during infections and vaccinations. It is plausible that CT and TT carriers might have an advantage in fighting infections; however, heightened CTL activity might also trigger unwanted autoimmune responses in these individuals. Finally, our novel unpublished observation that T allele is also significantly overrepresented in relapsing-remitting multiple sclerosis raise an important possibility that SIRPγ, together with other predisposing molecules such as HLA, might be playing a critical role in precipitating autoimmunity and warrants further studies.

## Material and Methods

### Human subjects

De-identified leukoreduction buffy coat samples from 79 healthy donors (HD) were obtained from the University of Iowa DeGowin Blood Center, Department of Pathology. The blood samples were a byproduct of platelet removal process. There were 42 females and 37 males with the range of 25–73. Informed consent was not required for the study. All studies were approved by the University of Iowa IRB according to Declaration of Helsinki principles.

### Cell preparation and bead sorting

PBMC were isolated from buffy coats using Ficoll Hypaque (GE Healthcare Biosciences, Pittsburg, PA) density gradient. PBMC samples and sorted cells were stored in freezing media in liquid nitrogen until further use in multiple assays. From PBMC preparations, purified CD8+, cells were isolated using Miltenyi microbead positive selection kits. Sorted CD8+ T-cells were subjected to flow sorting based on their SIRPγ expression. All magnetic microbeads were purchased from Miltenyi Biotech Inc. (Auburn, CA) and used according to manufacturer instructions, resulting in population purities >95%.

### Genotyping for rs2281808 detection

DNA was isolated from PBMC samples using Qiagen mini DNA prep kit. Allelic discrimination PCR was done using TaqMan assay and probe.

### mRNA expression analysis

RNA was isolated using Qiagen mini prep kit, reverse transcribed and quantitative PCR was performed using TaqMan assay. Samples were normalized to GAPDH.

### Flow cytometric antibody staining

Anti-human antibodies used for multi-color flow cytometric analysis included: CD3-Alexafluor 700, CD4-APC & PE-Cy7, CD8-BV786, SIRPγ-PE, CD45RO-Pacific Blue, CD27-FITC, CD127-APC, CD122-APC, CXCR3-APC, CD244-APC, T-bet-APC, Granzyme-APC. All antibodies were obtained from either BD Biosciences (San Jose, CA), Biolegend (San Diego, CA) or Miltenyi Biotech Inc. (Auburn, CA). PBMC samples were washed with 0.1% (w/v) sodium azide/phosphate-buffered saline (Mediatech Cellgro) and stained with fluorescently labeled anti-human antibodies, then resuspended in 1% paraformaldehyde (Electron Microscopy Sciences, Hatfield, PA). Intracellular cytokine staining was performed for T-bet and granzyme using eBioscience intracellular fixation and permeabilization buffer set. Flow cytometric data were acquired on 4-Laser LSRII using FACSDiva software (Becton Dickinson). Data were analyzed using Flow Jo (TreeStar, Ashland, OR).

### PBMC stimulation and cytokine detection

As described previously^19^, One million cells from HD were stimulated with PMA/Ionomycin/Brefeldin for 6 hours. Cells were washed with 0.1% (w/v) sodium azide/phosphate-buffered saline and stained intracellularly for detecting IFN-γ and TNF-α. In another assay, 1 million PBMC were stimulated with 0.1ug/ml, 0.5ug/ml or 1ug/ml anti-CD3 for 48 hours, followed by extracellular and intracellular staining for flow cytometry.

### RNA sequencing

SIRPγ^low^ and SIRPγ^high^ CD8+ T-cells from HD were subjected to RNA sequencing at University of Chicago Genomics facility. Sequencing was performed using an Illumina HiSeq. 2000 producing 50 bp single-end reads. Samples were aligned with the Kallisto pseudo-alignment^20^ and GRCh38 build for the human genome to produced estimated counts. Differential expression analysis was performed using tximport^21^ and DESeq2^22^ R packages to generate shrinkage estimates and fold changes using negative-binomial distributions and parametric modeling. Significant genes as defined as log2 fold-change greater than 1 and false-discovery rate of <0.05, were isolated from significant GSEA gene sets with FDR <0.25 in the C2 and C7 library^23^ related to the indicated pathway or process. Significant genes were then converted into z-scores and displayed in heatmaps. Figures were generated in GraphPad Prism v7 (La Jolla, CA)

### Statistical analysis

Data between the groups was analyzed with either 1-way or 2-way ANOVA with Tukey’s posthoc test and p < 0.05 was considered significant.

## Electronic supplementary material

Suppl Fig

## Data Availability

All data generated or analyzed during the study are included in this published article.

## References

[1] Barrett JC (2009). Genome-wide association study and meta-analysis find that over 40 loci affect risk of type 1 diabetes. Nature genetics.

[2] Kiani AK (2015). Association of 32 type 1 diabetes risk loci in Pakistani patients. Diabetes research and clinical practice.

[3] Reddy MV (2011). Association between type 1 diabetes and GWAS SNPs in the southeast US Caucasian population. Genes and immunity.

[4] Barclay AN, Brown MH (2006). The SIRP family of receptors and immune regulation. Nature reviews. Immunology.

[5] Piccio L (2005). Adhesion of human T cells to antigen-presenting cells through SIRPbeta2-CD47 interaction costimulates T-cell proliferation. Blood.

[6] Stefanidakis M, Newton G, Lee WY, Parkos CA, Luscinskas FW (2008). Endothelial CD47 interaction with SIRPgamma is required for human T-cell transendothelial migration under shear flow conditions *in vitro*. Blood.

[7] Gabrielsen IS (2016). Genetic risk variants for autoimmune diseases that influence gene expression in thymus. Human molecular genetics.

[8] Chowdhury FZ, Estrada LD, Murray S, Forman J, Farrar JD (2014). Pharmacological inhibition of TPL2/MAP3K8 blocks human cytotoxic T lymphocyte effector functions. PloS one.

[9] Knox JJ, Cosma GL, Betts MR, McLane LM (2014). Characterization of T-bet and eomes in peripheral human immune cells. Frontiers in immunology.

[10] McLane LM (2013). Differential localization of T-bet and Eomes in CD8 T cell memory populations. Journal of immunology.

[11] Fu SH, Yeh LT, Chu CC, Yen BL, Sytwu HK (2017). New insights into Blimp-1 in T lymphocytes: a divergent regulator of cell destiny and effector function. Journal of biomedical science.

[12] Kallies A, Xin A, Belz GT, Nutt SL (2009). Blimp-1 transcription factor is required for the differentiation of effector CD8(+) T cells and memory responses. Immunity.

[13] Shin H (2009). A role for the transcriptional repressor Blimp-1 in CD8(+) T cell exhaustion during chronic viral infection. Immunity.

[14] Zhou X, Xue HH (2012). Cutting edge: generation of memory precursors and functional memory CD8+ T cells depends on T cell factor-1 and lymphoid enhancer-binding factor-1. Journal of immunology.

[15] Zhou X (2010). Differentiation and persistence of memory CD8(+) T cells depend on T cell factor 1. Immunity.

[16] Kawasaki M (2009). Changes in the gene expression of peripheral blood mononuclear cells during the menstrual cycle of females is associated with a gender bias in the incidence of systemic lupus erythematosus. Clinical and experimental rheumatology.

[17] Joshi NS (2007). Inflammation directs memory precursor and short-lived effector CD8(+) T cell fates via the graded expression of T-bet transcription factor. Immunity.

[18] Westra, H. J. *Interpreting disease using functional genomics [S.I]:[S.n.]*, University of Groningen, (2014).

[19] Cunnusamy K (2014). Disease exacerbation of multiple sclerosis is characterized by loss of terminally differentiated autoregulatory CD8+ T cells. Clinical immunology.

[20] Bray NL, Pimentel H, Melsted P, Pachter L (2016). Near-optimal probabilistic RNA-seq quantification. Nature biotechnology.

[21] Soneson C, Love MI, Robinson MD (2015). Differential analyses for RNA-seq: transcript-level estimates improve gene-level inferences. F1000Research.

[22] Love MI, Huber W, Anders S (2014). Moderated estimation of fold change and dispersion for RNA-seq data with DESeq2. Genome biology.

[23] Mootha VK (2003). PGC-1alpha-responsive genes involved in oxidative phosphorylation are coordinately downregulated in human diabetes. Nature genetics.

