# Supplementary material for: An autoimmune disease risk SNP, rs2281808, in SIRPG is associated with reduced expression of SIRPγ and heightened effector state in human CD8 T-cells
Source: Sci Rep. 2018 Oct 18;8:15440. doi: 10.1038/s41598-018-33901-1 (PMC6194019; doi:10.1038/s41598-018-33901-1)
Supplement: Supplementary file 1 — Suppl Fig [file 41598_2018_33901_MOESM1_ESM.pdf]

# An autoimmune disease risk SNP, rs2281808, in SIRPG is associated with reduced expression of SIRPγ and heightened effector state in human CD8 T-cells

Sushmita Sinha[1]*, Nicholas Borcherding[1], Pranav S. Renavikar[1], Michael P. Crawford[1], Eva Tsalikian[2], Michael Tansey[2], Ezzatollah T. Shivapour[3], Frank Bittner[3], John Kamholz[3], Heena Olalde[3], Emilee Gibson[3] and Nitin J. Karandikar[1]

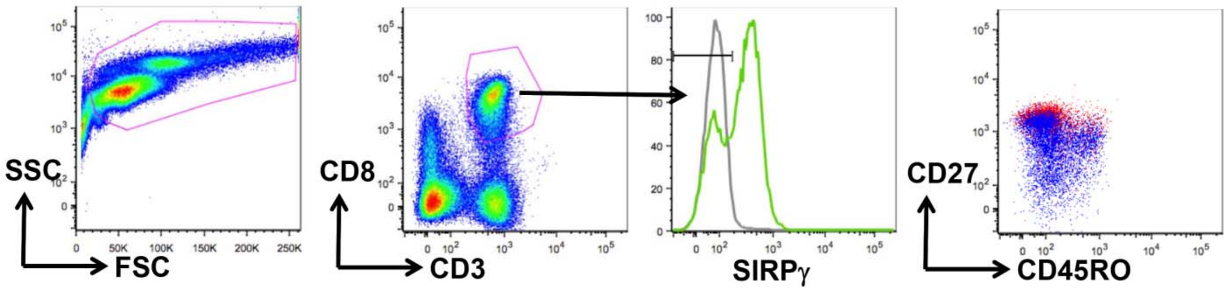


**Supplementary Fig 1**. Gating strategy for Fig 2. Live gated lymphocytes were gated on CD3+ CD8 T cells. SIRPγ expression on CD8 T cells was analyzed and SIRPγ low CD8 T-cells were gated. SIRPγ low CD8 T-cells were then overlaid over total CD8 T-cells displayed in the dot plot showing CD27 and CD45RO.

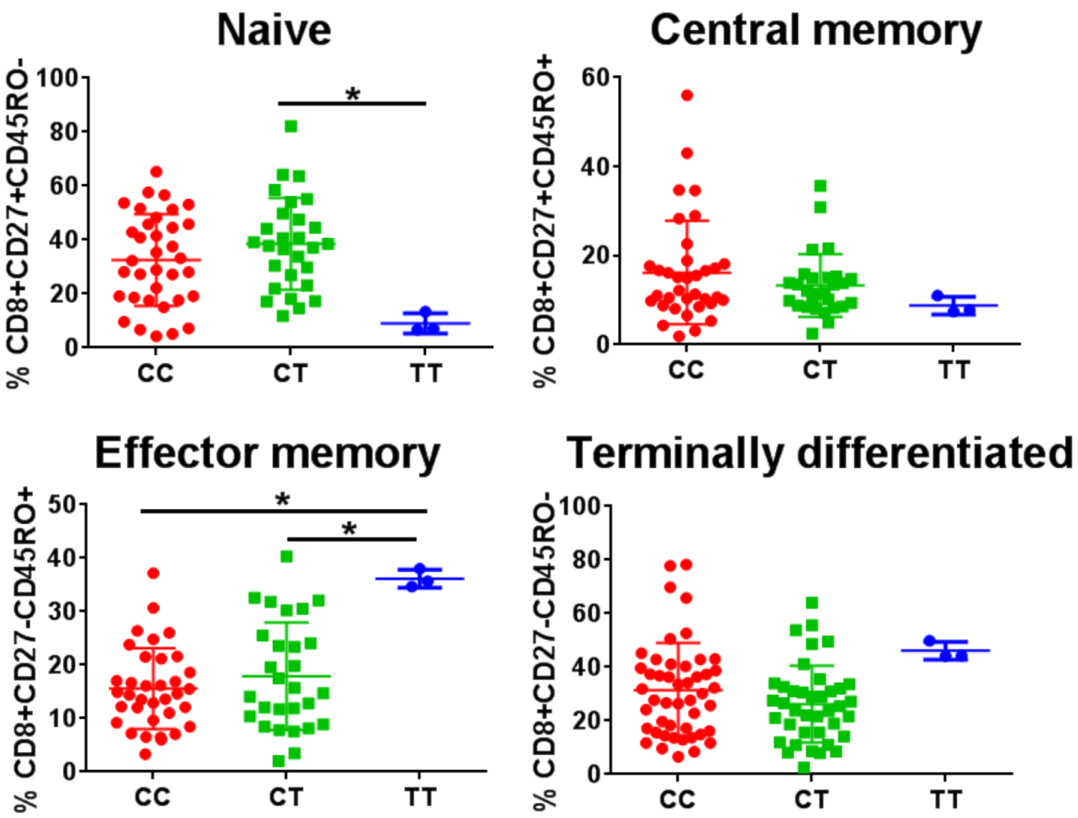


**Supplementary Fig 2**. CD8 T cell subsets with respect to rs2281808 genotype. PBMC samples were genotyped for rs2281808 by TaqMan PCR. PBMC samples were also subjected to flow cytometry staining. Gated CD3+CD8+ T-cells were analyzed for naïve (CD27+CD45RO-), central memory (CD27+CD45RO+), effector memory (CD27-CD45RO+), and terminally differentiated markers (CD27-CD45RO-) and plotted with respect to their rs2281808 genotype status. One-way ANOVA with Tukey's posthoc was performed and $p<0.05$ was considered significant.

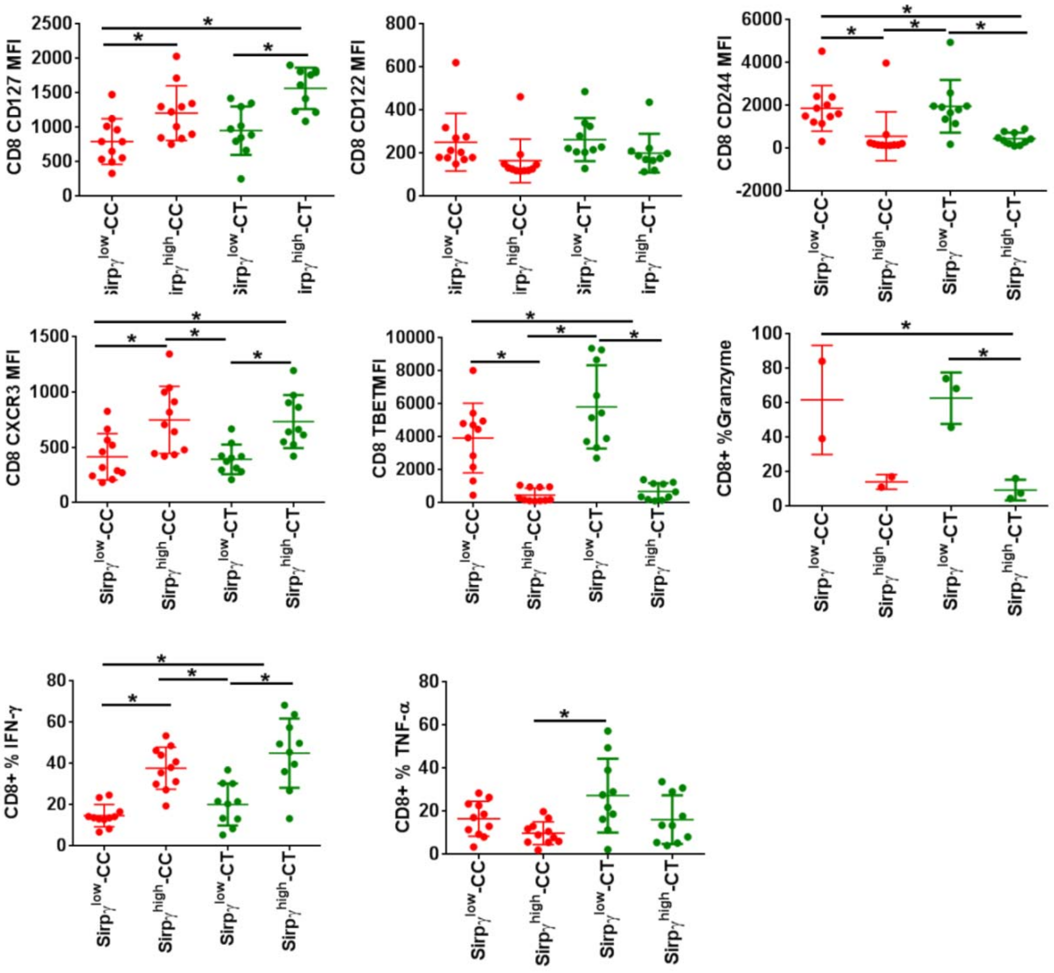


**Supplementary Fig 3**. Phenotypic profile of SIRPγ-high vs. low CD8 T-cells from CC and CT carriers are displayed separately. CD8 T-cells from CC, CT and TT carriers were evaluated for their expression of the indicated molecules by flow cytometry. Intracellular staining was performed for T-bet. In case of CC and CT, cells were gated for SIRPγ-high vs. low cells. PBMC samples from CC and CT carriers were stimulated with PMA/Ionomycin/brefeldin A for 5 hr before intracellular staining for IFN-γ and TNF-α. CD8 T-cells from CC and CT carriers were gated on SIRPγ$^{low}$ vs high cells for and evaluated for frequency of cells producing the indicted

cytokines. 1-way ANOVA with Tukey's posthoc analysis was performed and $p<0.05$ was considered significant.
